# Case report: Waldenstrom macroglobulinemia with systemic amyloidosis as the main manifestation

**DOI:** 10.3389/fmed.2024.1340553

**Published:** 2024-04-19

**Authors:** Junjing Yin, Xia Zhou, Shuyuan Yu, Hongying Wu, Yuping Zhong

**Affiliations:** ^1^School of Clinical Medicine, Qingdao University, Qingdao, China; ^2^Department of Hematology, Qingdao Municipal Hospital, Qingdao, China

**Keywords:** systemic amyloidosis, diarrhea, Waldenström macroglobulinemia, case report, lymphoplasmacytic cells

## Abstract

Systemic amyloidosis is a rare protein misfolding and deposition disorder leading to progressive organ failure. Waldenström macroglobulinemia (WM) with systemic amyloidosis as the main manifestation is even rarer. The patient in this study presented with recurrent diarrhea and had not been diagnosed in other hospitals on multiple occasions. Later, his diarrhea worsened and was accompanied by sunken edema of both lower limbs and dizziness. Renal biopsy showed deposits of PAS light-staining material in the glomeruli, interstitium, and small arteries, which stained positively with Congo red. Cardiac ultrasound showed interventricular septum thickening of 17 mm, right ventricular wall myocardial thickening of approximately 0.6 cm, and septal thickening of approximately 0.5 cm, considering myocardial amyloidosis. Electromyography showed abnormal peripheral nerve conduction. Lymphoplasmacytic cells were found in the bone marrow. Taken together, he was diagnosed with WM. He was treated with a BR (Bendamustine + Rituximab) regimen. After 6 courses, the patient’s discomfort was relieved, his weight gained 5 kg, the level of serum IgM and dFLC decreased, and cardiac and renal assessments were more relieved. The patient has been followed up for more than 1 month.

## Introduction

Waldenström macroglobulinemia (WM) is a rare B cell lymphoma, which accounts for <2% of all non-Hodgkin lymphomas. Hyperglobulinemia of monoclonal immunoglobulin M (IgM) protein is the hallmark of the disease (1). Recently, somatic mutation of the MYD88 gene has been reported in more than 90% of patients with WM (2). Clinical features include anemia, thrombocytopenia, hepatosplenomegaly, lymphadenopathy, and rarely hyperviscosity and amyloidosis (3). Systemic amyloidosis, as a rare manifestation of WM, mainly occurs in the kidney, heart, peripheral nerve, liver, and gastrointestinal tract and is caused by abnormal folding of the monoclonal IgM light chain, forming insoluble filaments and deposition (4). Therefore, patients can have non-specific symptoms, making initial diagnosis difficult. Herein, we describe a patient with WM characterized by systemic amyloidosis.

## Case report

A 52-year-old man was admitted to Qingdao Municipal Hospital in March 2023 as he had recurrent diarrhea for more than a year, accompanied by edema, dizziness, and limb numbness for several months. He presented with diarrhea as the initial presentation and was admitted to a community hospital in November 2022 with no definite diagnosis after a colonoscopy. Later, his diarrhea worsened and was accompanied by sunken edema of both lower limbs and dizziness, and he was treated in a superior hospital for further examination. A full blood count showed a hemoglobin concentration of 126 g/L, a white cell count of 6.85 × 10^9^/l, and a platelet count of 256 × 10^9^/l. Further blood tests showed a low albumin concentration of 26.9 g/L and an erythrocyte sedimentation rate of 56 mm/h. The urine test showed urinary protein 3+. Serum immunofixation electrophoresis proved the existence of monoclonal IgM and λ light chain. IgM was 16.38 g/L. The bone marrow biopsy showed that lymphocytes accounted for 10% of the total nucleated cells. Flow cytometric analysis indicated a population of monoclonal B lymphocytes accounted for 0.44% of the total nucleated cells in the bone marrow, and plasma cells accounted for 0.2%. These B lymphocytes were CD19 positive and CD5 dim in terms of immunophenotype. These plasma cells were CD38 positive, CD138 positive, CD19 dim, CD56 positive, and CD81 positive in terms of immunophenotype. The fluorescence in situ hybridization (FISH) results were negative for the deletion of the TP53 and RB-1 genes and negative for the rearrangement of the IGH gene. He also underwent a renal biopsy at another hospital, which showed deposits of PAS light-staining material in the glomeruli, interstitium, and small arteries that stained positively with Congo red. Immunofluorescence showed IgG linear+, IgA-, IgM-, C3-, C1q-, κ chain-, and λ chain+++. He underwent a cardiac ultrasound that showed interventricular septum thickening of 17 mm, right ventricular wall myocardial thickening of approximately 0.6 cm, and septal thickening of approximately 0.5 cm, considering myocardial amyloidosis. He was successively misdiagnosed by other hospitals with monoclonal gammopathy of undetermined significance (MGUS), monoclonal gammopathy of renal significance (MGRS), and primary systemic amyloidosis, and the treatment with methylprednisolone (40 mg × 7 d) was not effective.

His symptoms worsened, and he went to the Hematology Department of Qingdao Municipal Hospital in March 2023. On physical examination, he had multiple enlarged lymph nodes in his bilateral neck, supraclavicular, axillary, and inguinal regions. The largest lymph node was approximately 3 cm in diameter, tough, and non-tender, with good mobility and no adhesion to the surrounding tissues. He had mild sunken edema in both lower limbs, and he had lost 25 kg in the last 3 months. A full blood count showed normal results. His peripheral blood film showed marked red cell agglutination. Further blood tests showed a decrease in albumin concentration of 21 g/L, an increase in lactate dehydrogenase of 210.5u/l, β2-microglobulin of 2.08 mg/L, erythrocyte sedimentation rate of 37 mm/h, high-sensitivity troponin of 0.179 ng/mL, NT-ProBNP of 1704.98 pg./mL, and a prolongation of the activated partial thromboplastin time of 41.90 s. His calcium and renal profile were normal. Further studies on his immunoglobulin showed an IgG of 2.37 g/L, IgA of 0.71 g/L, and IgM of 13.10 g/L. The light chain in his urine was abnormal as well, with a κ light chain of 36.3 mg/L and λ light chain of 78.7 mg/L. Serum immunofixation electrophoresis proved the existence of monoclonal IgM and λ light chain. Serum-free light chain analysis showed a κ chain level of 8.89 mg/L, a λ chain level of 43.02 mg/L, a κ/λ of 0.2, and dFLC of 34.13 mg/L. M protein quantity was 10.4 g/L. His 24-h urine protein quantification was 8.5 g. Tests for rheumatism, including antinuclear antibody, rheumatoid factor, anti-double-stranded DNA antibody, and anti-SSA and SSB antibodies, were all negative. Serum tumor markers were also negative. Electromyography showed abnormal peripheral nerve conduction. PET-CT showed multiple lymph nodes in the V region of the right neck, left supraclavicular region, bilateral axilla, mediastinum, retroperitoneum, and bilateral inguinal area, some of which were slightly increased with tracer uptake, the diameter of the larger one was approximately 8.4 mm and SUV max of 1.6. Cardiac ultrasound showed a septal thickness of 17 mm, an enlarged left atrium, reduced left ventricular diastolic function, an E/A of 0.7, and an ejection fraction (EF) of 60%. ECG showed sinus rhythm and limb leads QRS wave hypopotentiation and ST-T morphologic changes.

**Figure 1:**
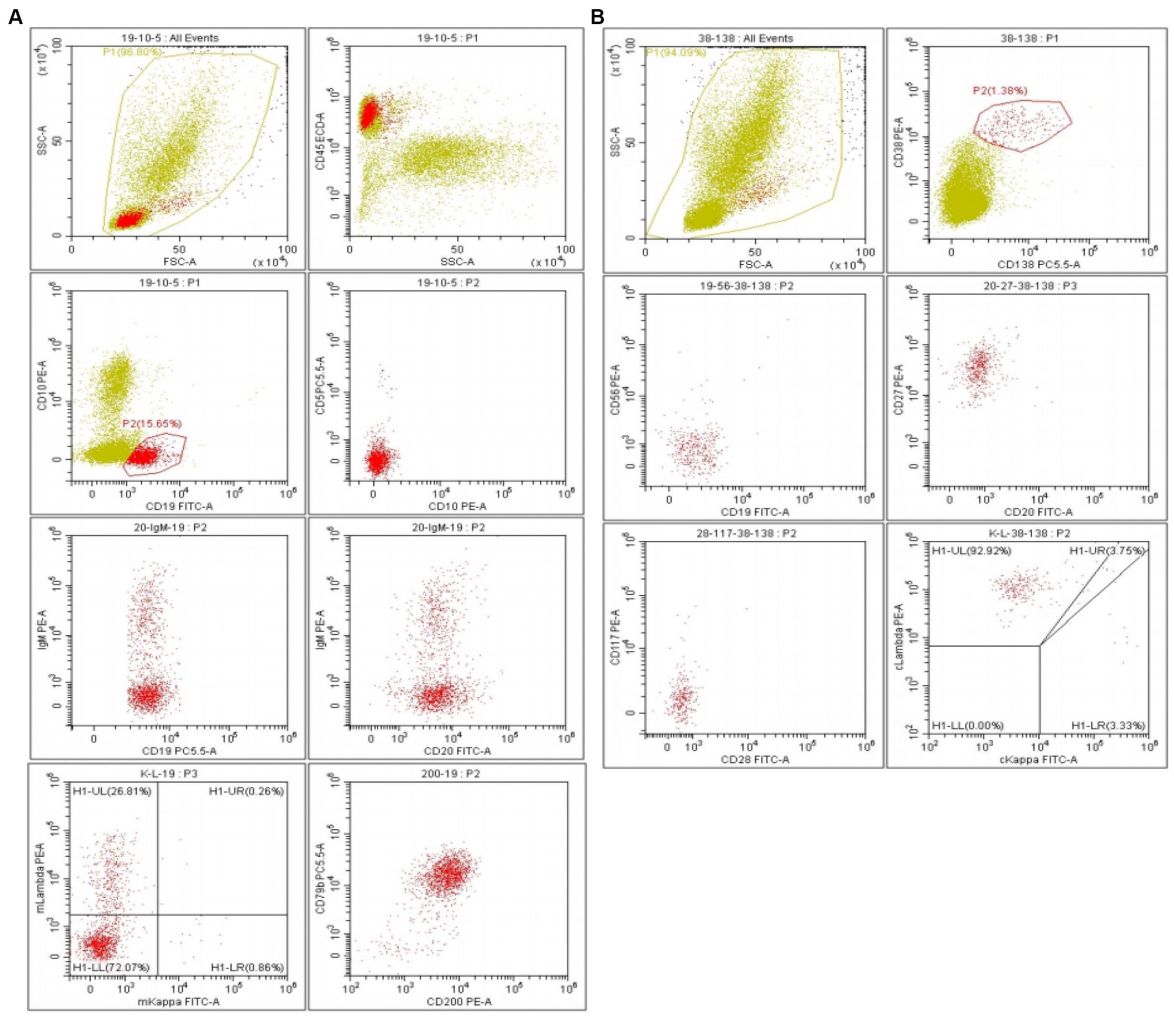
**(A)** Flow cytometric analysis indicated a population of monoclonal B lymphocytes accounting for 18.31% of total nucleated cells in bone marrow. **(B)** Monocolonal plasma cells accounted for 1.22%.

This patient underwent a repeat bone marrow biopsy, which showed that lymphocytes accounted for 26% of the total nucleated cells and 8% of plasma cells, and occasionally, lymphoplasmacytic cells were also seen. Flow cytometric analysis indicated that a population of monoclonal B lymphocytes accounted for 18.31% of total nucleated cells in the bone marrow, while plasma cells accounted for 1.22% (Figures 1A,B). The immunophenotypic profile for these B lymphocytes includes the expression of the pan B-cell antigens CD19, CD20, CD22, CD25, CD200, and CD79b, as well as the partial expression of the chain-restricted surface IgM and sLambda. These B lymphocytes have no expression of CD5, CD10, CD28, CD23, CD11c, and sKappa. These plasma cells include the expression of CD38, CD138, CD45, cLambda, CD19, and CD27 and have no expression of CD56, CD20, CD28, cKappa, and CD117 in terms of immunophenotype. Quantitative real-time PCR indicated a MYD88 L265P gene mutation with a mutation proportion of 0.442% (CD19 beads are not sorted). Taken together, he was diagnosed with WM. He was treated with a BR (Bendamustine + Rituximab) regimen. The treatment course lasted for 28 days. After 6 courses, his diarrhea and dizziness were significantly relieved, and he gained 5 kg. The patient underwent a comprehensive evaluation examination, IgM was 6.17 g/L, and a serum-free light chain analysis showed a κ chain level of 12.56 mg/L, a λ chain level of 26.91 mg/L, a κ/λ of 0.466, and dFLC of 14.35 mg/L. The 24-h urine protein quantification was 5,827 mg, and NT-ProBNP was 867.83 pg./mL. Cardiac ultrasound showed a septal thickness of 12.6 mm, the internal diameter of the remaining atrioventricular cavities was normal, ventricular systolic and diastolic function was normal, and EF was 60%. Imaging showed that the existing enlarged lymph nodes were smaller than before. There were no signs and symptoms of new disease activity.

## Discussion

The World Health Organization defines WM as a lymphoplasmacytic lymphoma associated with a monoclonal IgM protein (5). WM patients have different symptoms and complex manifestations. Patients with systemic amyloidosis are extremely rare and can easily be misdiagnosed or missed, which makes rapid diagnosis difficult. According to the guidelines for the diagnosis and management of WM published by the British Society of Haematology in 2022 (6), monoclonal IgM in serum and lymphoplasmacytic cells can be seen in the bone marrow of this patient. Combined with immunophenotype, WM can be diagnosed. This patient was easily misdiagnosed with IgM-MGUS because of the elevated serum monoclonal IgM. However, the patient with MGUS presented with no evidence of organ damage associated with plasma cells, except for the presence of monoclonal immunoglobulins (M proteins) (<30 g/L), and there were only a small number of mature plasma cells in the bone marrow rather than lymphoplasmacytic cells (7). The patient had obvious symptoms of diarrhea, dizziness, and fatigue, which were accompanied by multiple lymph node enlargements all over the body. Based on the bone marrow results, MGUS could be excluded. However, due to the edema of both lower limbs and proteinuria, the patient was easily misdiagnosed as having MGRS. MGRS is a spectrum of disorders in which renal injury is caused by M proteins secreted by benign or precancerous clonal B cells or plasma cells (8). By definition, this category does not meet the diagnostic criteria for multiple myeloma (MM) and WM and is used to describe patients who otherwise meet the diagnostic criteria for MGUS but who have M protein-induced kidney injury. MGRS mainly damages glomeruli, and the proportion of proteinuria is dominated by albuminuria rather than the light chain. In conclusion, the diagnosis of MGRS can be excluded in this patient. A renal biopsy confirmed renal amyloidosis, which should be differentiated from primary amyloidosis. Amyloidosis can be classified as primary or secondary depending on whether it is combined with hematologic tumors. The patient was weak and steadfastly refused a lymph node biopsy, and monoclonal B cells and monoclonal lymphocytes were seen in the patient’s bone marrow, with scattered lymphoplasmacytic cells. The MYD88 test was positive, so this patient was considered to have a diagnosis of WM with secondary amyloidosis, so it does not support the diagnosis of primary systemic amyloidosis.

Systemic amyloidosis is a rare disease involving multiple organs and causing organ dysfunction, with an annual incidence of 1/100000 ~ 9/100000 in Europe (9). It is a rare manifestation of WM, mainly in the kidney, heart, peripheral nerves, liver, and gastrointestinal tract, and its manifestations are non-specific and varied due to the variable location of amyloid deposits. Pathological biopsy shows positive Congo red staining and “apple green” birefringence under polarized light or fine fibers of 7–10 nm in diameter with no special morphology, disordered arrangement, and no branching under the electron microscope, and the diagnosis can be made if one of the above items is fulfilled (10). The patient presented with repeated diarrhea as the first symptom, which was aggravated later and accompanied by depressed edema of both lower extremities, dizziness and weakness, limb numbness, etc. He underwent a renal biopsy, which showed that the glomerulus, interstitium, and arterioles could be seen to be deposition of PAS-staining material, and the staining of Congo red was positive, and immunofluorescence has light chain λ-restricted expression. Cardiac ultrasound showed ventricular septum thickening to 17 mm, left and right ventricular walls thickened, right atrium enlarged, and EF of 60%, with myocardial amyloidosis considered. Electromyography showed abnormal peripheral nerve conduction, considering amyloidosis of the nervous system. Lymphoplasmacytic cells were seen in the bone marrow. Combined with the relevant laboratory indicators, it was considered that the patient, in this case, had systemic amyloidosis as the main manifestation of WM. Due to his kidney, heart, nervous system, gastrointestinal tract damage, and hypoproteinemia, poor prognosis was indicated (11, 12).

There is no uniform treatment standard for WM-associated amyloidosis; however, according to the WM guidelines issued by BSH in 2022 (6), this patient, who presents with combined multi-system amyloidosis, multiple enlarged lymph nodes, dizziness, and diarrhea, clearly has indications for treatment. The initial treatment regimen should be selected according to the patient’s age and tolerance, comorbidity, IgM quantity, tumor burden, MYD88L265P and CXCR4 gene mutation status, patient’s treatment willingness and economic ability, long-term treatment adherence, and efficacy of each regimen. Current first-line therapy includes rituximab alone or rituximab in combination with alkylating agents (bendamustine and cyclophosphamide), proteasome inhibitors (bortezomib and carfilzomib), nucleoside analogs (fludarabine and cladribine), and BTK inhibitors. BCL-2 inhibitors and stem cell transplantation can be used as salvage treatment options (13, 14). He suffered from multiple organ damage and obvious symptoms, so BR (Bendamustine + Rituximab) was selected for treatment. After 6 courses of treatment, his symptoms of diarrhea, dizziness, weakness, and limb numbness were significantly relieved, his weight gained 5 kg, and the level of serum IgM, 24-h urine protein, and NT-ProBNP were all decreased, and the follow-up effect required further follow-up observation.

## Conclusion

This case highlights a rare disease, presented with systemic amyloidosis (damage to the kidney, heart, gastrointestinal tract, and nervous system) and diarrhea as the first manifestation of the disease. The diagnosis should be made by combining the symptoms, signs, clinical manifestations, and auxiliary examination, and early treatment can improve the prognosis.

## Data availability statement

The original contributions presented in the study are included in the article/Supplementary material, further inquiries can be directed to the corresponding author.

## Ethics statement

Written informed consent was obtained from the individual(s) for the publication of any potentially identifiable images or data included in this article. Written informed consent was obtained from the participant/patient(s) for the publication of this case report.

## Author contributions

JY: Investigation, Writing – original draft, Writing – review & editing. XZ: Writing – review & editing. SY: Writing – review & editing. HW: Writing – review & editing. YZ: Supervision, Writing – review & editing.

## References

[1] McMaster ML. The epidemiology of Waldenström macroglobulinemia. Semin Hematol. (2023) 60:65–72. doi: 10.1053/j.seminhematol.2023.03.008, PMID: 37099032 PMC10351685

[2] Treon SP Xu L Guerrera ML Jimenez C Hunter ZR Liu X. Genomic landscape of Waldenström Macroglobulinemia and its impact on treatment strategies. J Clin Oncol. (2020) 38:1198–208. doi: 10.1200/jco.19.02314, PMID: 32083995 PMC7351339

[3] Gertz MA. Waldenström macroglobulinemia: 2023 update on diagnosis, risk stratification, and management. Am J Hematol. (2023) 98:348–58. doi: 10.1002/ajh.26796, PMID: 36588395 PMC10249724

[4] Palladini G Milani P. Diagnosis and treatment of AL amyloidosis. Drugs. (2023) 83:203–16. doi: 10.1007/s40265-022-01830-z 36652193

[5] Castillo JJ Advani RH Branagan AR Buske C Dimopoulos MA D'Sa S. Consensus treatment recommendations from the tenth international workshop for Waldenström Macroglobulinaemia. Lancet Haematol. (2020) 7:e827–37. doi: 10.1016/s2352-3026(20)30224-6, PMID: 33091356

[6] Pratt G El-Sharkawi D Kothari J D’Sa S Auer R McCarthy H. Diagnosis and management of Waldenström macroglobulinaemia—a British Society for Haematology guideline. Br J Haematol. (2022) 197:171–87. doi: 10.1111/bjh.18036, PMID: 35020191

[7] Gonsalves WI Rajkumar SV. Monoclonal Gammopathy of undetermined significance. Ann Intern Med. (2022) 175:ITC177–92. doi: 10.7326/aitc202212200 36508741

[8] Karam S Haidous M Dalle IA Dendooven A Moukalled N Van Craenenbroeck A. Monoclonal gammopathy of renal significance: multidisciplinary approach to diagnosis and treatment. Crit Rev Oncol Hematol. (2023) 183:103926. doi: 10.1016/j.critrevonc.2023.103926, PMID: 36736510

[9] Pinney JH Smith CJ Taube JB Lachmann HJ Venner CP Gibbs SD. Systemic amyloidosis in England: an epidemiological study. Br J Haematol. (2013) 161:525–32. doi: 10.1111/bjh.12286, PMID: 23480608 PMC4296340

[10] Goldis R Kaplan B Kukuy OL Arad M Magen H Shavit-Stein E. Diagnostic challenges and solutions in systemic amyloidosis. Int J Mol Sci. (2023) 24:4655. doi: 10.3390/ijms24054655, PMID: 36902083 PMC10003318

[11] Durot E Kanagaratnam L Zanwar S Kastritis E D'Sa S Garcia-Sanz R. A prognostic index predicting survival in transformed Waldenström macroglobulinemia. Haematologica. (2021) 106:2940–6. doi: 10.3324/haematol.2020.262899, PMID: 33179472 PMC8561274

[12] Kastritis E Morel P Duhamel A Gavriatopoulou M Kyrtsonis MC Durot E. A revised international prognostic score system for Waldenström's macroglobulinemia. Leukemia. (2019) 33:2654–61. doi: 10.1038/s41375-019-0431-y, PMID: 31118465

[13] Gertz MA. Waldenstrom Macroglobulinemia: tailoring therapy for the individual. J Clin Oncol. (2022) 40:2600–8. doi: 10.1200/jco.22.00495, PMID: 35700418 PMC9362871

[14] Ghafoor B Masthan SS Hameed M Akhtar HH Khalid A Ghafoor S. Waldenström macroglobulinemia: a review of pathogenesis, current treatment, and future prospects. Ann Hematol. (2023). doi: 10.1007/s00277-023-05345-9, PMID: 37414960

